# DipA, a Pore-Forming Protein in the Outer Membrane of Lyme Disease Spirochetes Exhibits Specificity for the Permeation of Dicarboxylates

**DOI:** 10.1371/journal.pone.0036523

**Published:** 2012-05-10

**Authors:** Marcus Thein, Mari Bonde, Ignas Bunikis, Katrin Denker, Albert Sickmann, Sven Bergström, Roland Benz

**Affiliations:** 1 Rudolf-Virchow-Center, DFG-Research Center for Experimental Biomedicine, University of Würzburg, Würzburg, Germany; 2 Department of Molecular Biology, Umeå University, Umeå, Sweden; 3 Department of Bioanalytics, Leibniz-Institut für Analytische Wissenschaften – ISAS, Dortmund, Germany; 4 School of Engineering and Science, Jacobs University Bremen, Bremen, Germany; Institute Pasteur, France

## Abstract

Lyme disease *Borreliae* are highly dependent on the uptake of nutrients provided by their hosts. Our study describes the identification of a 36 kDa protein that functions as putative dicarboxylate-specific porin in the outer membrane of Lyme disease *Borrelia*. The protein was purified by hydroxyapatite chromatography from *Borrelia burgdorferi* B31 and designated as DipA, for dicarboxylate-specific porin A. DipA was partially sequenced, and corresponding genes were identified in the genomes of *B. burgdorferi* B31, *Borrelia garinii* PBi and *Borrelia afzelii* PKo. DipA exhibits high homology to the Oms38 porins of relapsing fever *Borreliae*. *B. burgdorferi* DipA was characterized using the black lipid bilayer assay. The protein has a single-channel conductance of 50 pS in 1 M KCl, is slightly selective for anions with a permeability ratio for cations over anions of 0.57 in KCl and is not voltage-dependent. The channel could be partly blocked by different di- and tricarboxylic anions. Particular high stability constants up to about 28,000 l/mol (in 0.1 M KCl) were obtained among the 11 tested anions for oxaloacetate, 2-oxoglutarate and citrate. The results imply that DipA forms a porin specific for dicarboxylates which may play an important role for the uptake of specific nutrients in different *Borrelia* species.

## Introduction

Lyme disease is a systemic disorder manifested in a wide spectrum of different symptoms such as a circular skin rash around a tick bite and arthritis up to paralysis appearances and other neurological effects [1], [2]. It is caused by infection with Borrelia spirochetes [3], [4]. In Europe the main causative agents of Lyme disease include inter alia the species B. burgdorferi sensu stricto, B. garinii and B. afzelii [5]. Borreliae are obligate parasites and have a complex life cycle involving arthropod and mammalian reservoir hosts, usually ticks and rodents [6], [7]. To ensure the survival in this enzootic life cycle, the spirochetes must adapt to a range of diverse host environments and nutrient availability [8], [9], [10], [11], [12], [13]. Thus, these parasites need to have an efficient control of the nutrient uptake system across the cell envelope.

The *B. burgdorferi* cell envelope structure and outer membrane composition exhibit major differences as compared to those of other Gram-negative bacteria [14], [15], [16], [17], [18], [19]. For example, *B. burgdorferi* is known to lack lipopolysaccharides [20] and the flagella are localized in the periplasmic space [21]. In addition, the outer membrane has a low ratio of protein to lipid and a lower density than the inner membrane [19], [22], [23]. To date, a few integral membrane proteins have been identified and characterized in *B. burgdorferi* [18], [24], [25], [26], [27], [28]. Most proteins associated with the *Borrelia* outer membrane are lipoproteins [22], [29]. The few integral membrane proteins present in the *B. burgdorferi* outer membrane are therefore likely to act as pore-forming proteins.

Pore-forming proteins in Gram-negative and Gram-positive bacteria, so-called porins, are integral outer membrane proteins, which form large, water-filled pores in the outer membrane [30], [31] in order to enable the influx of nutrients and other substances from the environment into the bacterial cell. Porins can be subdivided into two classes: (i) general diffusion pores, such as OmpF of *E. coli* K12 [30,] which sort mainly according to the molecular mass of the solutes and (ii) pores with a binding site inside the channel. The latter porins are responsible for the rapid uptake of classes of solutes such as carbohydrates [32], [33], nucleosides [34] or phosphate [35]. Surface-exposed porin loops are potential targets for adhesion to other cells [36] as well as bacteriophages [37] and bactericidal compounds [38].

*Borrelia burgdorferi* has a relative small chromosome of 0.91 Mb, which is complemented by 21 linear and circular plasmids [39], [40], [41]. This small genome only codes for proteins of a few metabolic pathways. This means that *Borreliae* show a lack of biosynthetic capacity and their growth is dependent on a high diversity of nutrient compounds. One of those compounds described in this work are the dicarboxylates, which can have different roles in the bacterial physiology including amongst others production of energy, catabolism, respiration, basic/acid equilibrium and iron chelation [42].

Due to the limited metabolic capacities Borreliae are therefore highly dependent on nutrients provided by their hosts [40]. The important first step for the uptake of those nutrients into the bacterial cell is mainly limited by porins in the outer membrane. To date, two putative porins of *B. burgdorferi* have been characterized: P13 [43], [44], and P66 [27] with single-channel conductance of 3.5 nS, and 9.6 nS, respectively, in 1 M KCl. Another protein, formerly identified as porin Oms28 [45] was shown not to be a porin as it is localized in the periplasmic space [46]. Besides the porins, the channel-tunnel BesC, a TolC-homologue, which is a component of the *Borrelia* multi-drug-efflux systems, was identified in the outer membrane, forming channels of 300 pS in 1 M KCl [47].

**Figure 1:**
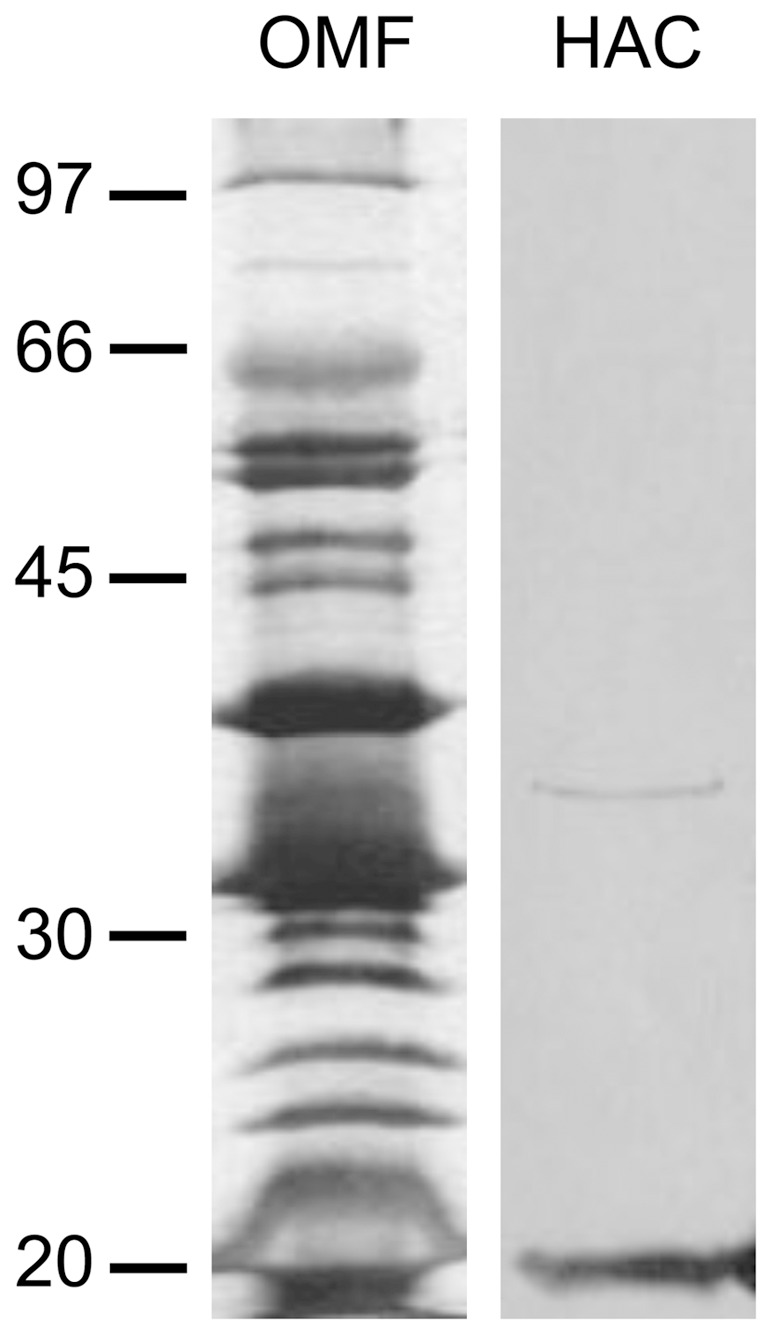
Analysis of purified DipA. Approximately 1–10 ng of outer membrane fraction (OMF) of *B. burgdorferi* B31 Δp66 or hydroxyapatite chromatography-purified (HAC) DipA was separated by 12% SDS-PAGE and silver-stained. The positions of molecular mass standards in kDa are shown at the left panel.

In this study, we report the purification and biophysical characterization of a dicarboxylate-specific porin in the outer membrane of *B. burgdorferi*, a homologue of the Oms38 porin of relapsing fever spirochetes [48]. Subsequently, homologous proteins of this newly identified porin are present in the Lyme disease agents *B. garinii* and *B. afzelii*, sharing a high amino acid homology of 88%. The pore-forming protein was purified by hydroxyapatite chromatography and designated as DipA, for dicarboxylate-specific porin A. Study of the protein using the black lipid bilayer method revealed anion selectivity of the channel that has a conductance of 50 pS in 1 M KCl. DipA is the first identified solute-specific porin in *Borrelia*. It contains at least one binding site with a high affinity for dicarboxylic anions and related compounds and is therefore suggested to play a major role in the metabolic pathway for the uptake of these nutrients.

## Results

### Purification and identification of a new pore-forming protein in the outer membrane of *B. burgdorferi Δp66*

The outer membrane fraction (OMF) of B. burgdorferi B31 Δp66 contains a variety of proteins as shown by SDS-PAGE (Fig. 1, left panel). Previous black lipid bilayer experiments with OMFs of B. burgdorferi Δp66 and B. burgdorferi Δp13/Δp66 [49] indicated that the preparations contained high channel-forming activities in the conductance range between 10 and 100 pS which are not related to P13, Oms28, P66 and BesC. Also the recent identification of an 80 pS-porin in closely related relapsing fever spirochetes suggested the possible presence of a similar pore-forming protein in the B. burgdorferi OMF [48]. To identify the corresponding protein component, approximately 100 µg of the OMF of B. burgdorferi Δp66 [49], a knock-out mutant of the 11 nS pore P66, was subjected to hydroxyapatite chromatography. The fraction eluted at an ionic strength of 250 mM KCl showed high channel-forming activity of 50 pS in 1 M KCl which differed clearly from the previously described pore-forming activities of P13 [43], Oms28 [44], P66 [27] and BesC [46]. To check the purity of the protein fraction exhibiting the channel formation, 100 µl of the corresponding fraction were precipitated and subjected to a 12% SDS-PAGE. Pore formation was found exclusively in fractions containing a band that corresponded to a molecular mass of 36 kDa (Fig. 1, right panel).

**Figure 2:**
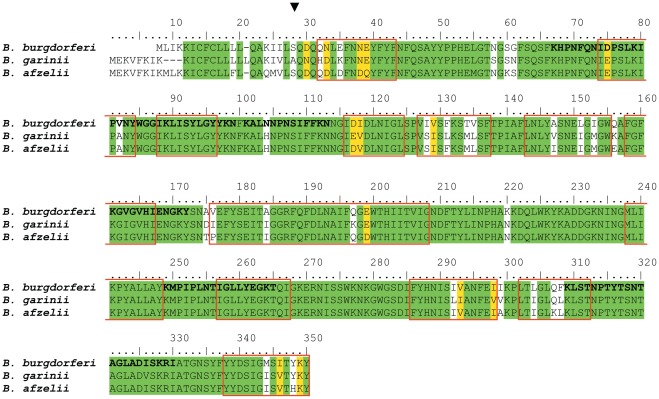
Amino acid sequence alignment of DipA of B. *burgdorferi* B31 (B.b.), *B. garinii* PBi (B.g.) *B. afzelii* PKo (B.a.) and B. duttonii (B.d.). The alignment was performed using Pole Bioinformatique Lyonnaise Network Protein Sequence Analysis (http://npsa-pbil.ibcp.fr). Amino acids identical in all four proteins are highlighted in red, strongly similar amino acids (:) are given in green and weakly similar ones (.) in blue. The putative beta strands in DipA of *B. burgdorferi* are indicated by blue bars as derived from secondary structure prediction programs [55], [56]. The cleavage site of the N-terminal signal peptide of DipA of *B. burgdorferi* B31 as predicted by the program SignalP 3.0 (http://www.cbs.dtu.dk/services/SignalP/) with maximum probability (about 50%) is marked by a black bar [54]. This is the same site as has been found previously for the N-terminal end of Oms38 of *B. duttonii* by N-terminal sequencing [48]. Partial peptide sequences obtained by mass spectrometry are in bold and highlighted in yellow.

To identify the gene coding for this 36 kDa protein, silver-stained protein bands of the SDS-PAGE gel were tryptically digested, analyzed by mass spectrometry and identified by peptide mass fingerprinting. The fraction eluting from the hydroxyapatite column at an ionic strength of 250 mM contained besides the 36 kDa protein a second band visible through all fractions, which corresponded to a molecular mass of about 20 kDa. Mass spectrometry identified this band as truncated versions of OspA and OspB, well-known cell surface proteins, which are present in very high copy numbers in the OMF of *B. burgdorferi* (Fig. 1). Detailed molecular and structural analyses of OspA and OspB [50], [51], [52] and the fact that fractions next to one with pore-forming activity contained exclusively the OspA/OspB band and did not exhibit pore-forming activity suggested that these proteins did not form pores. Thus, the 36 kDa protein was clearly defined as sole pore-forming component in the hydroxyapatite fraction. It was responsible for the formation of the 50 pS pores. The gene coding for the protein band with the apparent molecular mass of 36 kDa was identified as “hypothetical protein *bb0418*” (GenBank accession number NP_212552) of *B. burgdorferi* B31, now designated as *dipA*, for dicarboxylate-specific porin A. The partial peptides identified by mass spectrometry are marked in figure 2. Searches within the published genomes of *B. garinii* PBi and *B. afzelii* PKo revealed homologous genes to *dipA* in these closely related Lyme disease agents.

### Analysis of the amino acid sequences of DipA of *B. burgdorferi*, *B. garinii* and *B. afzelii*

The DipA sequences of *B. burgdorferi*, *B. garinii* and *B. afzelii* are shown together with that of *B. duttonii* in figure 2. DipA of the first three species share an amino acid sequence identity of 88% demonstrating that the proteins are highly conserved. Most of their sequence heterogeneity is found in the N-terminal region. Strikingly, *B. burgdorferi* DipA is 57% identical with the Oms38 porin of the relapsing fever species *B. duttonii* (see figure 2) [48], which exhibited similar biophysical properties (see below). As known from other spirochetal outer membrane proteins N-terminal amino acids serve as signal peptides and are cleaved under *in vivo* conditions [53]. N-terminal cleavage sites of *B. burgdorferi*, *B. garinii* and *B. afzelii* DipA as predicted by the program SignalP 3.0 (http://www.cbs.dtu.dk/services/SignalP/) with maximum probability of about 50% are marked by a black mark [54]. It is noteworthy that the predicted N-terminal cleavage sites of these three proteins agree well with that of Oms38 of *B. duttonii* (see figure 2) derived from N-terminal amino acids sequencing [48]. Further computational analysis [55], [56] predicted putative ß-strands (blue bars in figure 2), that suggested that approximately 45–50% of the secondary structure of DipA may consist of ß-sheets similar as is known for the ß-barrel cylinders of well-studied bacterial porins [57], [58].

### Immunoblot analysis of outer membranes and purified *B. burgdorferi* DipA

**Figure 3:**
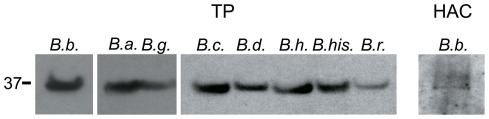
Detection of DipA in Lyme disease and relapsing fever spirochetes. Immunoblot analysis with antiserum against *B. burgdorferi* DipA resulted in clear signals of DipA and its homologues in the total protein fractions (TP) of the Lyme disease agents *B. burgdorferi* (*B. b.*), *B. afzelii* (*B. a.*), *B. garinii* (*B. g.*) and the relapsing fever agents *B. crocidurae* (*B. c.*), *B. duttonii* (*B. d.*), *B. hermsii* (*B. h.*), *B. hispanica* (*B. his*) and *B. recurrentis* (*B. r.*). The immunoblot signal of the hydroxyapatite chromatography (HAC) purified *B. burgdorferi* (*B. b.*) DipA is on the right. The position of molecular mass standard in kDa is shown at the left.

For immunoblot analysis antiserum was raised against a recombinant polypeptide representing the 90 C-terminal amino acids of *B. burgdorferi* DipA. Using this antiserum immunoblots of the total protein fractions (TP) of different Lyme disease and relapsing fever *Borrelia* and a fraction containing purified *B. burgdorferi* DipA were performed (Fig. 3). The results demonstrated that polyclonal anti-DipA serum clearly detected DipA in the TP of the Lyme disease species *B. burgdorferi*, *B. afzelii* and *B. garinii*. Furthermore, the immunoblot showed also strong signals within the TP of the relapsing fever species *B. crocidurae*, *B. duttonii*, *B. hermsii*, *B. hispanica* and *B. recurrentis*, which indicated cross-reactivity of the anti-DipA polyclonal serum with the DipA homologue Oms38 [48]. In addition, we found also a signal within the hydroxyapatite chromatography fraction that showed pore-forming activity (Fig. 3, HAC).

### Localization of DipA

**Figure 4:**
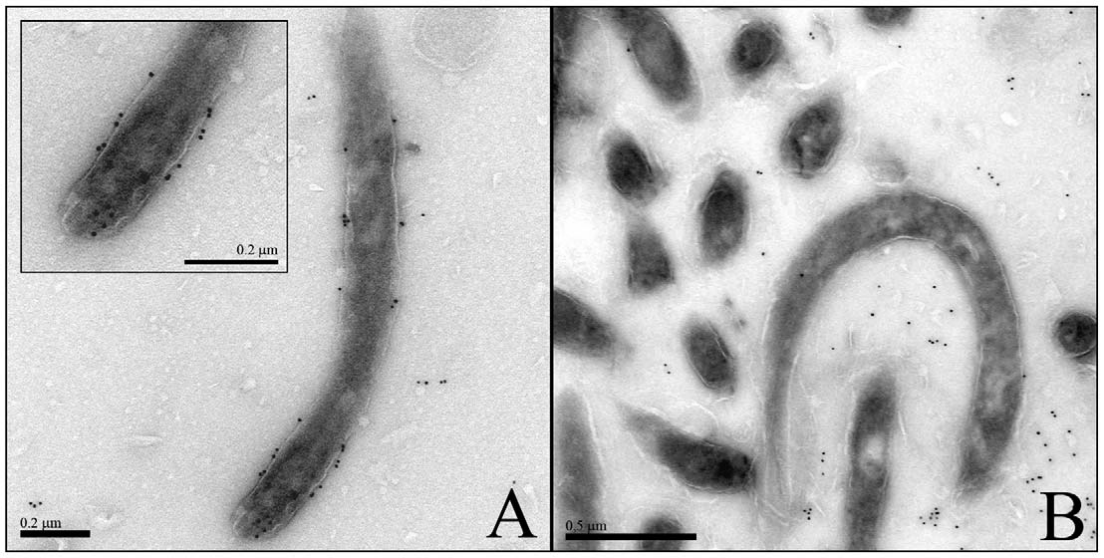
Transmission electron micrograph showing immunogold labeled DipA in the outer membrane of the *B. burgdorferi* Osp-less mutant B313. Ultrathin cryosections were prepared from the *B. burgdorferi* Osp-less mutant B313 at −110°C, embedded in gelatin. The immungold particles were visualized by sequential incubation of the fixed cells by the polyclonal rabbit antibodies detecting (A) DipA or (B) pre-serum and the anti-rabbit antibodies GAR10.

The presence of an N-terminal cleavage site of DipA and the enrichment of DipA in outer membrane fractions strongly indicated the outer membrane localization of the protein. To further demonstrate this we performed sequential incubation of Osp-deficient *B. burgdorferi* B313 cells with DipA antibodies or pre-immune serum and gold-labeled GAR10 detecting rabbit antibodies. The Osp-deficient mutant was used to better visualize outer membrane proteins other than the Osp-proteins [59]. Electron micrographs of cryosectioned *B. burgdorferi* B313 cells stained with anti-DipA antiserum showed immunogold particles on the spirochetal outer surface (figure 4 A). No immunogold particles could be seen on spirochetes stained with pre-immune serum and the anti-rabbit antibodies GAR10 (figure 4 B). It is noteworthy that the immunogold particles were only visible in the region of the envelope of *B. burgdorferi* cells. This means that the antigenic structure is indeed localized on the surface of the *B. burgdorferi* cells, i.e. DipA is an outer membrane protein.

### Single-channel experiments

**Figure 5:**
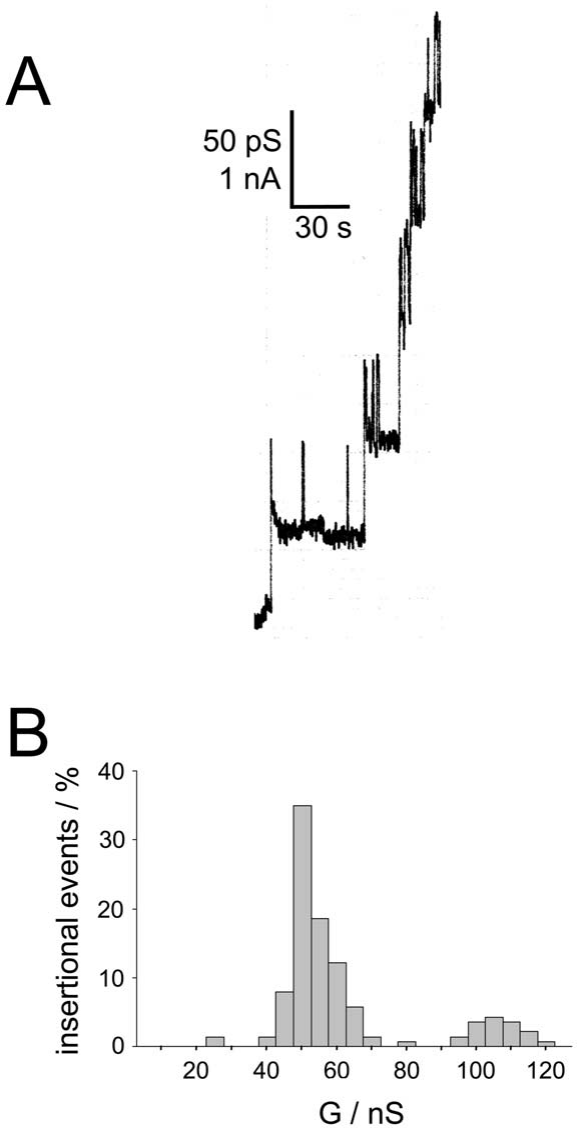
Pore-forming activity of DipA. (A) Single-channel conductance observed for DipA in a diphytanoyl phosphatidylcholine/*n*-decane (PC) membrane. About 10 ng ml^−1^ of purified DipA of about was added to a PC lipid bilayer, bathed in 1 M KCl. (B) Histogram of individual single-channel events observed for purified DipA. The average single-channel conductance was 50 pS for a total number of 140 single steps; temperature  = 20°C; voltage  = 20 mV.

DipA-mediated channel formation was studied in detail. The addition of small amounts of DipA to a black lipid bilayer membrane caused a substantial conductance increase due to the formation of small ion-permeable channels similar to pore-forming events caused by other bacterial porins [30]. Under conditions of appropriate amplification and low protein concentration, the recording of single reconstitution events into the membrane could be resolved as conductance steps with an average single-channel conductance of 50 pS in 1 M KCl (figure 5A). Figure 5B shows a histogram of the current fluctuations observed with DipA in 1 M KCl. The data suggested that the current fluctuations are rather homogeneous but show a considerable noise level that may limit the accuracy of our single-channel data. Interestingly, the 50 pS channel-forming activity of DipA could be completely abolished after preincubation with DipA-specific polyclonal rabbit antiserum, which also demonstrated antibody binding to DipA and that the channels were definitely caused by this protein (data not shown).

Single-channel experiments were also performed with other electrolytes such as LiCl and KCH_3_COO to obtain more information on the properties of the channels formed by DipA. By statistical analysis of at least 100 conductance steps, the single-channel conductance of DipA was evaluated as a function of different electrolytes and different concentrations. The results are summarized in Table 1 and suggested anion selectivity of the channel. There was some influence of the mobility of anions on conductance (45 pS in 1 M KCH_3_COO, pH 7), whereas change of the cation did not influence conductance (50 pS in 1 M LiCl). Table 1 shows also the average single-channel conductance of DipA, *G*, as a function of the KCl concentration in the aqueous phase. The single channel conductance in different KCl concentrations was a linear function of the electrolyte concentration.

**Table 1:** Average single-channel conductance (*G*) of DipA in different electrolyte solutions.

Electrolyte	Concentration	*G*
	(*M*)	(*pS*)
KCl	0.1	8
	0.3	20
	1	50
	3	140
LiCl	1	50
KCH_3_COO (pH 7)	1	45

The membranes were formed from diphytanoyl phosphatidylcholine dissolved in *n*-decane. The aqueous electrolyte solutions were unbuffered and had a pH of ∼6 unless otherwise indicated; temperature  = 20°C; voltage  = 20 mV. The average single-channel conductance, *G*, was calculated from at least 30 single reconstitution events of DipA.

### Voltage dependence

Some Gram-negative bacterial porins show voltage-dependent closure despite the fact that no voltage dependent closure was observed so far in *in vivo* experiments [30], [60], [61]. A multi-channel experiment with at least 100 reconstituted DipA channels was performed to check the protein for a possible voltage-dependence. The application of membrane potentials ranging from −120 V to +120 V did not show any influence on the conductance demonstrating that DipA did not show voltage-dependent closure in the tested voltage range.

### Selectivity measurements

Selectivity measurements were performed to quantify the permeability of the DipA channel for anions relative to cations. The selectivity was checked by multi-channel experiments under zero-current potential conditions. Membranes were formed in 100 mM electrolyte solution and purified DipA was added to the aqueous phase when the membranes were in the black state. After incorporation of at least 100 channels into the membrane, five-fold salt gradients were established across the membranes by addition of small amounts of 3 M salt solution to one side of the membrane. The zero-current potential on the more diluted side of the membrane was negative for KCl (−10.1 mV) and LiCl (−11.9 mV), suggesting preferential movement of anions through the DipA channel for these salts (Table 2). In contrast, the zero-current membrane potential was positive (7.5 mV) using KCH_3_COO as electrolyte, suggesting preferential movement of potassium over acetate ions. The permeability ratios of cations over anions through DipA were calculated from the zero-current potentials using the Goldman-Hodgkin-Katz equation [62]. They revealed together with the zero-current membrane potential that DipA is preferentially anion selective, because the ratios of the permeability coefficients P_cation_/P_anion_ were 0.57 (in KCl), 0.47 (in LiCl) (Table 2). The P_cation_/P_anion_ in KCH_3_COO was 1.65, which means that also cations could have certain permeability through DipA. Furthermore the selectivity of the channel for anions in KCl could be influenced (P_cation_/P_anion_  = 0.79) by adding 1 mM oxaloacetate (in 10 mM Tris-HCl pH 7.5) to the KCl solution. Oxaloacetate can interact with the pore (see below), and the decreased anion selectivity indicates that addition of oxaloacetate (and its possible binding too the channel) can influence the characteristics of the channel, e.g. its selectivity.

**Table 2:** Zero-current membrane potentials (*V*_m_) of diphytanoyl phosphatidylcholine/*n*-decane membranes in the presence of DipA measured for a five-fold concentration gradient of different electrolytes.

Electrolyte	*V*_m_	*P*_c_/*P*_a_
	(*mV*)	
KCl	−10.1	0.57
LiCl	−11.9	0.47
KCH_3_COO (pH 7)	7.5	1.65
KCl + 1 mM Oxaloacetate	−7.6	0.79

*V*_m_ is defined as the difference between the potential at the dilute side (100 mM) and the potential at the concentrated side (500 mM). The aqueous electrolyte solutions were buffered with 10 mM Tris-HCl (pH 7.5); temperature  = 20°C; The permeability ratio *P*_c_/*P*_a_ was calculated using the Goldman-Hodgin-Katz equation [62] from at least three individual experiments.

### Partial blockage of ion flux through DipA by addition of dicarboxylates

Single-channel measurements demonstrated that DipA formed very small pores with a conductance much smaller than that of typical general diffusion pores [31]. The small single-channel conductance and the fact that growth of *Borrelia* is highly dependent on the uptake of certain nutrients [40], [63] suggested that DipA could be a channel specific for essential nutrients and contained a binding site for them in a similar way as the carbohydrate-specific *E. coli* channel LamB [32], [33].

To test this hypothesis, titration experiments using different classes of substrates were performed as described previously for titration of LamB with carbohydrates [32], [64]. Interestingly, most classes of substrates including carbohydrates, such as glucose, fructose, sucrose, maltose and lactose, nucleosides, such as adenosine, and other anionic molecules, like acetate, carbonate, phosphate and adenosine triphosphate, did not show any interaction with DipA. However, partial channel block was observed for dicarboxylates, which was studied in detail. For these experiments an electrolyte was chosen containing 0.1 M KCl, 1 mM Tris-HCl, pH 7.5, close to the chloride concentration in the blood of mammals to work under almost physiological conditions. This means that the experiments were performed at a pH at least 1 unit above the p*K*_s_ of the carboxylic groups in the aqueous phase to guarantee dissociation of carboxylic groups of the dicarboxylates by at least 90%. DipA was reconstituted into lipid bilayer membranes. After reconstitution of channels had slowed down considerably and the membrane conductance was approximately stationary, concentrated solutions of different dicarboxylates were added to the aqueous phase at both sides of the membrane while stirring to allow equilibration. To exclude conductance decrease caused by pH and dilution effects during the addition of certain solutes, all tested substrates were dissolved in 0.1 M KCl, 1 mM Tris-HCl, pH 7.5 and the conductivity of the bathing solution was checked before and after each titration experiment.

**Figure 6:**
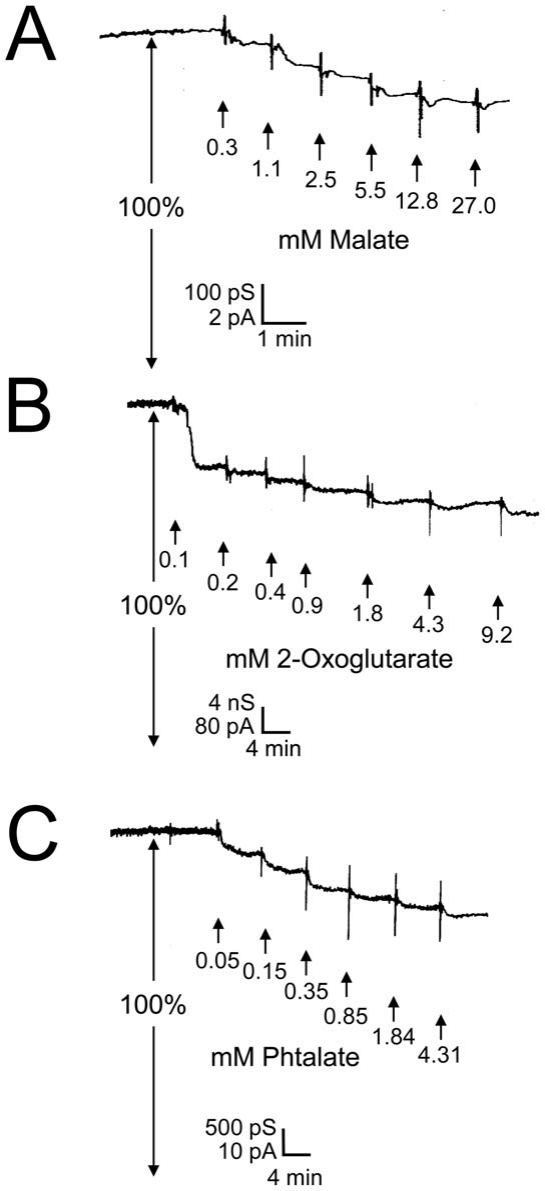
Titration of membrane conductance induced by DipA with malate (A), 2-oxoglutarate (B) and phthalate (C). The membrane was formed from diphytanoyl phosphatidylcholine/*n*-decane. The aqueous phase contained ∼100 ng ml^−1^ DipA protein, 0.1 M KCl, 10 mM Tris-Cl, pH 7.5 and dicarboxylates at concentrations as indicated; temperature  = 20°C; voltage  = 20 mV.

Fig. 6 shows experiments using malate (Fig. 6A), 2-oxoglutarate (Fig. 6B) and phthalate (Fig. 6C) as potential substrates of DipA. The addition of these dicarboxylates led to a dose-dependent block of DipA-mediated membrane conductance, which decreased by 23% in the case of malate, 29% in the case of 2-oxoglutarate and 25% in the case of phthalate at substrate concentrations of 27 mM, 9 mM and 4 mM, respectively. To study the complete binding potential of DipA for the substrates, titration experiments were performed with a variety of dicarboxylates and other related organic anions with high biological relevance (Table 3). All anions listed in this Table blocked the ion current through DipA with a maximum block of channel conductance ranging from 20% for pyruvate to 31% for oxaloacetate.

**Table 3:** Stability constants, *K*, for the binding of different organic anions to the DipA channel.

Organic anion	*K*	*K*_s_	maximum inhibition of channel conductance	*n*	
	(*l/mol*)	(*mM*)	(*%*)		
*Dicarboxylic anions (C4)*					
Oxaloacetate	19,900±5,100	0.05±0.01	31		5
Succinate	6,100±2,200	0.18±0.06	24		2
Malate	1,300±520	0.87±0.33	23		2
*Stereospecific*					
Fumarate	420±38	2.42±0.22	28		2
Maleate	28,300±950	0.04±0.00	23		2
*Dicarboxylic anion (C5)*					
2-Oxoglutarate	3,500±140	0.35±0.16	29		3
*Aromatic dicarboxylic anion (C8)*					
Phthalate	5,700±710	0.18±0.02	25		2
*Tricarboxylic anion (C6)*					
Citrate	13,000±2,700	0.08±0.02	24		2
*Other substrates*					
Aspartate	1,300±450	0.82±0.33	27		3
Glutamate	1,250±590	0.90±0.43	22		2
Pyruvate	470±34	2.12±0.15	20		2

The organic anions are important key metabolites of *Borrelia* species. The membranes were formed from diphytanoyl phosphatidylcholine dissolved in *n*-decane. The buffered aqueous phase (1 mM Tris-HCl pH 7.5) contained purified DipA in a concentration of about 100 ng/ml and 0.1 M KCl; temperature  = 20°C; voltage  = 20 mV. The stability constants were derived from titration experiments similar to those shown in Fig. 3. The stability constant, *K*, is given as the mean of *n* experiments ± SD. *K*_s_ is the half-saturation constant.

### Study of the binding affinity of different dicarboxylates to DipA

**Figure 7:**
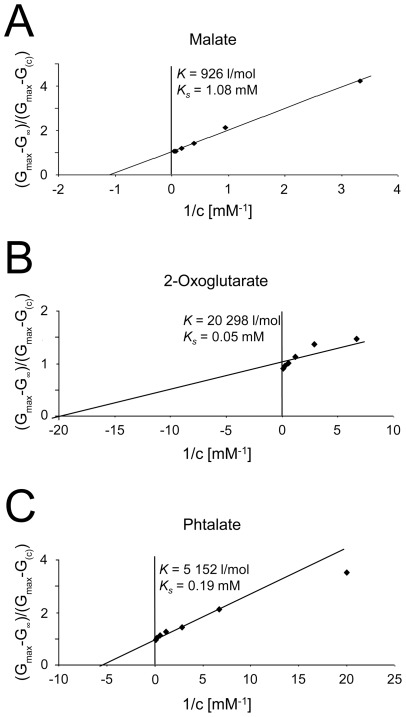
Lineweaver-Burk plots of the inhibition of membrane conductance by malate (A), 2-oxoglutarate (B) and phthalate (C). The data were taken from figure 6 and analyzed using equation 4. The straight lines correspond to stability constants *K*, for malate binding of 926 l/mol (*K*_s_ = 1.08 mM), for 2-oxoglutarate 20,298 l/mol (*K*_s_ = 0.05 mM) and for phthalate 5,152 l/mol (*K*_s_ = 0.19 mM).

The titration experiments with DipA were analyzed in a similar way as used for the characterization of carbohydrate-binding channels of Gram-negative bacteria [32], [64]. The data of figure 6 and of similar experiments were analyzed using equation 4, which means that Lineweaver-Burke plots were performed as shown in figure 7 for the data of figure 6. The straight lines in figure 7 corresponded to stability constants, *K*, for malate 930 l/mol (*K*_s_ = 1.1 mM) (Fig. 7A), for 2-oxoglutarate 20,300 l/mol (*K*_s_ = 0.05 mM) (Fig. 7B) and for phthalate 5,150 l/mol (*K*_s_ = 0.19 mM) (Fig. 7C).

Table 3 summarizes the results of all titration experiments. Binding of dicarboxylic anions yielded high stability constants for oxaloacetate (*K* = 19,900±5,100 l/mol), succinate (*K* = 6,100±2,200 l/mol), malate (*K* = 1,300±520 l/mol) and 2-oxoglutarate (*K* = 3,500±140 l/mol). This means that binding of the tested compounds to the DipA channel showed a significant specificity. C_4_-dicarboxylates with terminal groups next to one of the carboxylic groups showed considerable differences in their stability constants. The binding constant was maximal for oxaloacetate (*K* = 19,900±5,100 l/mol) which contains a polar oxo group next to one of the carboxylic groups. Succinate, a dicarboxylate without any side groups, showed a significantly lower binding affinity (*K* = 6,100±2,200 l/mol). For malate and aspartate, which contain a hydroxyl group and a positively charged amino group, respectively, next to the carboxyl acid groups, the stability constants were even smaller (*K* = 1,300±520 l/mol and 1,300±450 l/mol, respectively).

The use of the unsaturated C_4_-dicarboxylic anions fumarate and maleate yielded a stability constant for fumarate (*K* = 420±38 l/mol) that was remarkably low. The *trans* position of the carboxylic groups seemed to reduce significantly the affinity to the binding site of DipA. In contrast, the stability constant of maleate with a *cis* position of the carboxylic groups allowed maximum binding interaction resulting in a drastic increase of the stability constant (*K* = 28,300±950 l/mol). Experiments with 2-oxoglutarate demonstrated that an increase of the carbon chain length of the C_4_ oxaloacetate to a C_5_-dicarboxylic anion affected the binding affinity again drastically and resulted in a decrease of the stability constant from 19,900±5,100 l/mol (oxaloacetate) to 3,500±140 l/mol (2-oxoglutarate). Phthalate, an aromatic C_8_-dicarboxylic anion, exhibited a stability constant of 5,700±710 l/mol indicating that binding of larger compounds is still possible. Interestingly, the use of bulkier dicarboxylates did not result in a higher maximum inhibition of channel conductance, which was also 25% for phthalate and in the same range of block than for the other dicarboxylates tested here.

Titration experiments with citrate revealed a high stability constant (*K* = 13,000±2,700 l/mol), pointing out that a third carboxylic group in the organic anions leads to further increase of the binding affinity compared to 2-oxoglutarate that lacks the third acid group and has a polar oxo group instead. For aspartate and glutamate, containing positively charged amino groups next to one of the carboxylic groups, the stability constants were relatively low (1,300±450 l/mol and 1,250±590 l/mol, respectively), indicating a certain influence of the positively charged amino group on the binding affinity. For the monocarboxylic C_3_-anion pyruvate the observed binding affinity was very low (*K* = 470±34 l/mol) and comparable to the value of fumarate (*K* = 420±38 l/mol).

Taking all results of the binding affinities together, the DipA channel showed rather high stability constants in the range from 420 l/mol to 28,300 l/mol for a wide spectrum of organic anions containing one, two or three carboxylic acid groups. The highest stability constants were measured for C_4_-dicarboxylic anions such as maleate and oxaloacetate. Furthermore, these results revealed distinctive binding specificities of DipA to certain substrates depending on the number of carboxylic acid groups and on side groups of the anions like oxo, hydroxyl or amino groups.

## Discussion

### Identification of *B. burgdorferi* DipA

Using hydroxyapatite chromatography, DipA could be purified from the OMF of *B. burgdorferi* in the same way as the purification of the DipA homologue Oms38 of relapsing fever *Borrelia* [48]. SDS-PAGE analysis revealed a high degree of purity of DipA after protein precipitation and silver-staining of the gel. In addition to the identification using mass spectrometry and immunoblot analysis clearly confirmed that the 36 kDa band in the outer membrane of *B. burgdorferi* is the DipA protein responsible for pore formation. This could further be demonstrated by block of its pore-forming ability by preincubation with antiserum against DipA. Presumably, the specific antiserum bound to DipA and blocked its reconstitution and thus its pore-forming capacity.

The 20 kDa protein band additionally visible on SDS-PAGE after purification across the hydroxyapatite column is definitely not responsible for pore-formation because fractions next to that with pore-forming activity contained exclusively this 20 kDa band and did not exhibit any pore-forming activity. This band could clearly be identified by mass spectrometry as truncated versions of OspA and OspB, well-studied outer surface proteins of *B. burgdorferi* [50], [51], [52], [65], [66]. Thus, previous detailed molecular and structural analyses of these proteins supported our view that they are lipoproteins without any channel-forming ability.

### Amino acid sequences of *B. burgdorferi*, *B. garinii* and *B. afzelii* DipA

The deduced amino acid sequences of the three LD species' DipA share an identity of 88%, which means that the identities between the DipA homologues are very high, comparable with the high homology of other porins in these species [24], [25]. From this point of view we assumed that structure and function of the DipA homologues are identical under *in vivo* conditions. Strikingly, *B. burgdorferi* DipA is also 57% identical to the porin Oms38 of the relapsing fever (RF) agent *B. duttonii* [48]. This high amino acid identity is in agreement with immunoblot results, which showed that DipA-specific antiserum reacted with analogous protein domains of DipA and Oms38 in total protein fractions of both LD and RF *Borrelia*. In addition, similar biophysical characteristics (see below) of DipA and the Oms38 underlined this finding on the amino acid level and suggested that these proteins are homologues in agents of Lyme disease and in agents of relapsing fever.

DipA is located in the outer membrane meaning that this protein needs to contain an N-terminal signal peptide with a putative recognition sequence for the leader peptidase similar to those of the homologous relapsing fever porin Oms38 of *B. duttonii*, which has been N-terminally sequenced [48], and other spirochetal outer membrane proteins [53]. The predicted signal peptide for *B. burgdorferi* DipA is 20 amino acids long and contains positive charges at the N-terminus, properties which are typical for borrelial signal peptides [53]. Further predictions indicated that the deduced sequences of *B. garinii* and *B. afzelii* DipA contain similar N-terminal extensions that are responsible for their transport into the periplasm as is known for other spirochetal porins [53].

The secondary structure predictions supported the idea that the proteins may form a β-barrel cylinder consisting of about 14 β-sheets. However, the β-sheet predictions could be tentative and only 3D-crystallography can give a final answer. This is characteristic for all known Gram-negative bacterial porins, which form β-barrel cylinders containing 16 or 18 β-sheets [30], [57], [67], [68].

### Biophysical properties of DipA

DipA was characterized using artificial diphytanoyl phosphatidylcholine (PC) bilayers. PC is also present in the outer membrane of *B. burgdorferi* in a relation of about 1∶1 together with phosphatidylglycerol (PG) and glycolipids as major lipid components, which comprise about 50% of the total lipids of the outer membrane [69], [70]. Phosphatidylethanolamine (PE) and lipopolysaccharides (LPS) which are both typical gram-negative bacterial lipids were not found in *B. burgdorferi* outer membranes [69], [71]. Preliminary experiments using PG membranes did not show differences to the use of PC membranes. Reconstitution experiments with PC membranes and DipA allowed a meaningful comparison with other bacterial and borrelian pore-forming proteins, which have been studied under identical conditions: the single-channel conductance of 50 pS differs clearly from the comparatively high single-channel conductance of 600 pS [45], 3.5 nS [44] and 9.6 nS [27] of the other *B. burgdorferi* porins and from the BesC channel tunnel (300 pS) [46]. Nevertheless, the DipA pore showed a small single-channel conductance comparable to the ones of the substrate-specific *E. coli* channels Tsx (10 pS) [72] and LamB (160 pS) [32] under identical conditions. The reconstitution and biophysical properties of DipA were similar to the ones observed for the homologue Oms38 [48]. Congruently with Oms38, during some single-channel measurements initial sharp peaks and superposition of the stable 50 pS state of the pore (see Fig. 5) have been observed, which could be interpreted as additional transient states of the DipA channels. An anion selectivity of the DipA channels was indicated by single-channel measurements in LiCl and KCH_3_COO and could be confirmed by zero-current potential measurements. Interestingly, the selectivity for anions could be reduced by the addition of 1 mM oxaloacetate to the KCl solution, which presumably bound to the channel and resulted in a partial shielding of exposed charges. This result suggested the possibility that DipA could contain a binding site for dicarboxylates.

### Specificity of DipA for dicarboxylates

The growth of *Borrelia* depends strictly on nutrients provided by their hosts as demonstrated by the fastidious growth requirements of serum-supplemented mammalian tissue-culture medium for *in vitro* cultivation [63]. In addition, it is known that *B. burgdorferi* lacks genes coding for proteins of the tricarboxylic acid cycle or oxidative phosphorylation and for *de novo* synthesis of amino acids and nucleotides [40]. This implicates that essential compounds or precursor of these compounds have to be imported into the cell.

Porins with a similar small single-channel conductance as DipA often contain specific substrate-binding sites [32], [33], [34], [35], which suggested that DipA could possibly be a substrate-specific porin of *B. burgdorferi*. This assumption was tested and the substrate-specificity could be demonstrated by multi-channel experiments which revealed that DipA has a high affinity for dicarboxylates. Despite the observed high affinity for these organic acids, it was not possible to measure the permeability of DipA for these metabolites. Anyway, in analogy to other bacterial specific porins, it is likely that the DipA binding site with its high affinity for dicarboxylic anions increases the permeability of the channel for these metabolites as has been demonstrated previously: The presence of a binding site leads to an accelerated transport of carbohydrate through LamB and of phosphate transport through OprP, especially at very low substrate concentrations [30], [64]. Thus, the permeability of a substrate-specific porin can surpass that of a general diffusion pore by orders of magnitude in spite of its smaller cross-section [64].

Dicarboxylates, such as malate, succinate, oxaloacetate and 2-oxoglutarate, are major intermediates of the tricarboxylic acid cycle and mainly used for synthesis of amino acids. In addition, C_4_-dicarboxylates other than succinate can be metabolized due to the lack of a functional tricarboxylic acid cycle in anaerobic energy metabolism of most bacteria 42]. Taking these points into consideration, a potential dependence of the growth of *Borrelia* on this group of chemicals is likely. This hypothesis is additionally supported by the fact that the serum-supplemented mammalian tissue-culture medium for *in vitro* cultivation of *Borreliae* is supplemented by pyruvate and the tricarboxylic citrate. Amongst others, these compounds have been shown to specifically bind to DipA.

Consequently DipA plays an important role in the uptake of dicarboxylates and related compounds across the outer membrane. It is noteworthy that DipA is not the first identified membrane channel that is specific for dicarboxylates. Previous studies revealed that the channel of spinach leaf peroxisomes is also specific for this class of chemicals [73]. Interestingly, in bacteria, the PorB porin of *Chlamydia trachomatis* is the first identified pore-forming outer membrane protein being specific for dicarboxylates [74]. The detailed study of the DipA specificity revealed that the stability constants depended strongly on the specific structure of the organic anion showing a maximum for C_4_-dicarboxylates. Even if the observed stability constants are low compared to those of other substrate-specific bacterial porins such as LamB or Tsx of *E. coli* [34], [64] they are in the same range as values observed for the binding of dicarboxylates to the channel of spinach leaf peroxisomes [73] and higher than stability constants of specific porins for nicotinamide adenosine dinucleotide and nicotinamide mononucleotide (NAD and NMN). Even under saturated substrate concentrations the channel conductance of DipA could be maximally blocked by 30%, which means that there are still ions able to pass the pore as known from other porins and the dicarboxylates-specific channel of peroxisomal membranes [73]. Even phthalate, which is much bigger than the other tested chemicals, could not lead to a complete block of the channel conductance. This could indicate that the binding site is not localized in the interior of the channel but at the entrance in a binding pocket or at a surface-exposed loop. However, it is also possible that the binding site is in a pocket in the interior of the channel. Occupation of the binding site should in such a case not completely block ion transport.

The binding site would lead to increased concentrations of dicarboxylates in the close proximity of the pore and therefore to an accelerated uptake. Our data suggested together with the observed anion selectivity that the DipA binding site consists of positively charged groups. Taking these findings together, a porin could be identified in the outer membrane of *B. burgdorferi*, designated as DipA. DipA does not form general diffusion pores, but represents a specific porin. Its permeability properties are determined by charge effects of a permeability filter. Thus, DipA is the first identified *Borrelia* porin exhibiting a substrate specificity and therefore has presumably a well-defined function. Interestingly, despite several attempts by us to delete DipA in *B. burgdorferi*, so far this has not been successful, indicating that this deletion might be lethal for the cells. This study supplements the knowledge of the outer membrane protein composition of LD species and could lead to a basis for a successful drug design, more information concerning the physiology of the spirochetes and discover a surface-exposed protein that could function as a potential vaccine candidate.

## Materials and Methods

### Bacterial strains and growth conditions

The Lyme disease strains used in this study were *B. afzelii* [76], *B. garinii* LU185 [77] and *B. burgdorferi* strains B31 (ATCC 35210) and *B. burgdorferi* Δp66, a *p66* knock-out strain of *B. burgdorferi* B31-A [49] and the Osp-deficient *B. burgdorferi* B313 [78]. The relapsing fever bacterial isolates used were *B. crocidurae* CR2 (from the strain collection of Alan G Barbour UC Irvine), *B. duttonii* 1120 [79], *B. hermsii* (ATCC35209), *B. hispanica* CR1 [79] and *B. recurrentis* A1 [59]. Bacteria were grown in Barbour-Stoenner-Kelly-II (BSKII) medium [63] supplemented with 10% rabbit serum and 1.4% gelatin at 37°C until cell density reached approximately 10^7^–10^8^ cells ml^−1^ followed by harvesting the cells by centrifugation.

### Isolation of outer membrane proteins and purification of the 36 kDa protein

Outer membrane fractions (OMFs) of *B. burgdorferi* Δ*p66* used in this study were prepared as described elsewhere [80]. Purification of the native porin was performed by using a hydroxyapatite Bio-gel (Bio-Rad) column as it has been used previously for the purification of mitochondrial porins [81], [82] and the porin Oms38 of relapsing fever spirochetes [48]. 100 μl of OMF (approx. 100 µg proteins) were dissolved in 400 μl 2% Genapol (Roth). The mixture was applied to a hydroxyapatite column made from 0.3 g hydroxyapatite in an Econo-Column (Bio-Rad) with the dimensions of 0.5×5 cm and a column volume of 2 ml. The column was washed with six column volumes of a buffer containing 2% Genapol, 10 mM Tris-HCl (pH 8.0). For protein elution four column volumes of a buffer containing 2% Genapol, 250 mM KCl and 10 mM Tris-HCl (pH 8.0) were passed through the column. Fractions of 2.0 ml volume were collected.

### SDS-PAGE and Immunoblotting

Sodium dodecyl sulfate-polyacrylamide gel electrophoresis (SDS-PAGE) was performed according to the Laemmli gel system [83]. 100 µl of hydroxyapatite-chromatography fractions were precipitated by the protocol of Wessel and Flügge [84]. Proteins were separated by 12% SDS-PAGE under denatured conditions (boiled for 5 min in 4x SDS sample buffer before loading the gel) by using a Bio-Rad electrophoresis system. The gels were silver-stained [85]. For immunoblots, a tank blot system (Amersham Biosciences) was used as previously described [86]. Bound antibodies were detected using peroxidase-conjugated anti-rabbit or anti-mouse antibodies (DAKO A/S) and enhanced chemiluminescence reagents according to the manufacturer's instructions (Amersham Biosciences).

### Overexpression of a recombinant fragment of DipA

A fragment of DipA representing the 90 C-terminal amino acids was produced in *E. coli* Rosetta™ 2 (DE3) (Novagen), using expression vector pET 15b (Novagen) containing an N-terminal His_6_-Tag. The gene fragment of 285 bp was amplified by PCR using following oligonucleotides: rbb0418_f (5′-CTG**CATATG**GAAGGAAAAACACAAATTGG-3′) containing NdeI restriction site and rbb0418_r (5′-GACTTTA**GGATCC**TTAAGTTATAGACATTCC-3′) containing BamHI restriction site. After restriction enzyme digestion, the PCR product was ligated into the plasmid pET-15b. The *E. coli* cells carrying expression plasmids were grown at 37°C to OD_600_  = 0.6 in LB medium containing 50 µg of carbecillin per ml and protein expression was induced by addition of isopropyl-β-d-thiogalactopyranoside (IPTG) to a final concentration of 1 mM. The culture was further grown for 4 h, and cells were collected by centrifugation at 6,000× *g* for 15 min. The cells were lysed using BugBuster 10X Protein Extraction Reagent (Novagen) according to manufacturer's instructions. Recombinant fragment containing an N-terminal His-Tag was purified using Ni-NTA Spin Columns (Qiagen) following manufacturer's recommendations. Elution fractions were combined and proteins were precipitated using trichloroacetic acid (TCA). Briefly, to protein solution TCA was added to a final concentration of 5%, samples were incubated on ice for 30 min., pelleted by centrifugation, washed with cold acetone, pelleted and resuspended in NuPAGE® LDS Sample Buffer (Invitrogen).

### Antiserum

Rabbit polyclonal antiserum was raised against the recombinant fragment of DipA produced as described above. Precipitated elution fractions were separated by SDS-PAGE electrophoresis. Recombinant protein fragment was excised from the gel and approximately 100 µg of protein was used for rabbit immunization and subsequent boosts (Agrisera AB, Sweden).

### Mass spectrometry

The hydroxyapatite fractions showing pore-forming activity were subjected to SDS-PAGE followed by silver-staining [85]. The two bands were analyzed by mass spectrometry (nano LC-MS/MS) as described elsewhere [87]. Data of the MS/MS datasets were evaluated by Mascot algorithm [88]. In detail, mass spectrometric analysis was performed on a Qtrap4000 linear ion trap system (Applied Biosystems, Darmstadt, Germany). Mass spectra obtained by LC-MS/MS analysis were used to identify the corresponding peptides with the MascotTM (version 2.1.6) [88]. The algorithm searched in the Uniprot *Borrelia* FASTA database (April, 2007) with the following parameter set: (a) fixed modification: carbamidomethyl (C); (b) variable modification: oxidation (M); (c) peptide and MS/MS tolerance: +/− 0.4 Da; (d) ion score cut-off: 30.

### Preparation of *B. burgdorferi* B313 for transmission electron microscopy and cryo-EM imaging

The spirochetes were initially washed in PBS supplemented with 5 mM MgCl_2_ and pelleted by centrifugation at 3,000× g for 20 minutes. For fixation the bacteria were resuspended in 2% paraformaldehyde in 0.1 M phosphate buffer (pH 7.4). After 2 h, the fixative was removed and the bacteria were washed with PBS, PBSGly (glycine 0.15%) and finally pelleted in 10% gelatin in phosphate buffer. The gelatin was allowed to solidify, and small cubes were cut at 4°C and infused with 2.3 M sucrose for at least 2 h at 4°C. The blocks were mounted on a specimen holder and frozen in liquid nitrogen. Ultrathin cryosections were prepared at −110°C on a Leica EM UC7/EM FC7 (Leica, Vienna, Austria) with a diamond knife. The polyclonal rabbit anti-DipA antiserum was affinity-purified and used as the primary antibody for the immunostaining of the spirochetes. Pre-immune serum served as negative control. Immunogold labeling was performed by the method of Slot *et* *al.* (1991) [89] by sequential incubation of the polyclonal rabbit antibody detecting DipA (1∶50 in PBS) and GAR10 (1∶20) (BBI, England). The sections were examined in a Jeol 1230 TEM. Digital images were capture by using a Gatan MSC 600CW.

### Planar lipid bilayer assay

The methods used for black lipid bilayer experiments have been described previously [90]. The instrumentation consisted of a Teflon chamber with two compartments separated by a thin wall and connected by a small circular hole with an area of about 0.4 mm^2^. The membranes were formed from a 1% (w/v) solution of diphytanoyl phosphatidylcholine (PC) (Avanti Polar Lipids, Alabaster, AL) in *n*-decane. The porin-containing protein fractions were 1∶100 diluted in 1% Genapol (Roth) and added to the aqueous phase after the membrane had turned black. The membrane current was measured with a pair of Ag/AgCl electrodes with salt bridges switched in series with a voltage source and a highly sensitive current amplifier (Keithley 427). The temperature was kept at 20°C throughout. To analyze the effect of DipA-specific antibodies on channel-forming abilities of DipA, preincubation with antibodies was performed as previously described [91]. Briefly, approximately 100 ng of purified DipA was incubated with polyclonal antiserum against DipA in a ratio of 1∶3, incubated for 1 h at room temperature, and investigated in the planar lipid bilayer assay.

Zero-current membrane potential measurements were performed by establishing a five-fold salt gradient across membranes containing approximately 100 pore-forming proteins as it has been described earlier [62], [92]. The zero-current membrane potentials were measured with a high impedance electrometer (Keithley 617). Voltage-dependence of the porin channels was checked following the method described elsewhere [93], using membrane potentials as high as −120 to +120 mV.

Binding of dicarboxylates to DipA was investigated in the same way as the binding of maltooligosaccharides to carbohydrate-specific porins [32], [64]. Binding of the substrate to a binding site inside the channel could be detected by a reduced ion flux through the channel. These measurements were performed with multi-channel experiments under stationary conditions. The protein was added to black diphytanoyl phosphatidylcholine/*n*-decane membranes. The membrane conductance increased upon reconstitution of channels. After about 90 minutes the conductance was stationary. At that time dicarboxylates were added in defined concentrations to both sides of the membrane while stirring continuously to allow equilibration. Compounds bound to the DipA channel resulted in a dose-dependent decrease of the membrane conductance as result of the restricted ion flux. The conductance data of the titration experiments were analyzed using the following equations [64]. The conductance, *G(c)*, of a DipA channel in the presence of dicarboxylates with the stability constant *K* (half saturation constant *K_S_*) and the dicarboxylate concentration, *c*, is given by the maximum conductance (without dicarboxylates), *G_max_*, times the probability that the binding site is free:
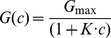
(1)


Equation (1) may also be written as
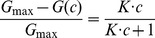
(2)which means that the conductance as a function of the dicarboxylate concentration can be analyzed using Lineweaver-Burke plots.

Equation (2) did not provide a satisfactory fit of the data from titration experiments, a fact which could be explained by the assumption that the DipA channels did not close completely when they were occupied by the different compounds or that only a fraction of the DipA channels closed completely. As previously described, equation (3), which took this problem into account, allowed a much better fit [75],
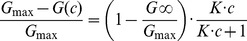
(3)


Equation (3) can also be written as:
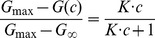
(4)where *G*_∞_ is the conductance at very high substrate concentration, i.e. the fraction of the conductance that did not respond to the block of the channels by dicarboxylate compounds.
